# The Genetic Foundations of Serotonin Syndrome, Neuroleptic Malignant Syndrome, and Malignant Hyperthermia: Is There a Genetic Association Between These Disorders?

**DOI:** 10.7759/cureus.10635

**Published:** 2020-09-24

**Authors:** Juan Fernando Ortiz, Martín Wirth, Noha Eskander, Jazmin Carolina Cozar, Olumuyiwa Fatade, Bindu Rathod

**Affiliations:** 1 Neurology, California Institute of Behavioral Neurosciences & Psychology, Fairfield, USA; 2 Neurology, Universidad San Francisco de Quito, Quito, ECU; 3 Psychiatry, California Institute of Behavioral Neurosciences & Psychology, Fairfield, USA; 4 Medicine, Universidad de las Americas, Quito, ECU; 5 Gynecology, Open Door Family, Portchester, USA; 6 Psychiatry and Behavioral Sciences, Hackensack Meridian Jersey Shore University Medical Center, Neptune, USA; 7 Psychiatry and Behavioral Sciences, California Institute of Behavioral Neurosciences & Psychology, Fairfield, USA

**Keywords:** neuroleptic malignant syndrome, serotonin syndrome, malignant hyperthermia

## Abstract

Neuroleptic malignant syndrome (NMS), serotonin syndrome (SS), and malignant hyperthermia (MH) share similar clinical characteristics. These conditions can present life-threatening situations due to exposure to different drugs. A similar genetic predisposition is suspected between these syndromes as well. This review aims to consolidate the knowledge about the genetics of these disorders and find possible correlations among them to frame the best possible approaches using different drugs without producing life-threatening complications that can be preventable. As a method, we collected data using PubMed with a Medical Subject Headings (MeSH) strategy. The inclusion criteria were as follows: full papers, studies conducted on humans, papers published in the English language, and study types that included case reports, journal articles, multicenter studies, clinical studies, observational studies, or clinical trials. Studies involving animals, articles that were without a visible abstract, study types that included clinical reviews, systematic reviews, or meta-analyses were excluded. 146 papers were reviewed, and 130 papers were removed for no possible extraction of data, duplication of the data, or the study outcome was not compatible with the objective of this review. Ultimately, a total of 17 papers were used for the discussion of this article. As a result of this review, we found no genetic association between NMS, SS, and MH development. Finally, we conclude that NMS, SS, and MH presentation are caused by different mutations which are not associated. ^3^However, because of the life-threatening clinical presentation of these conditions, genetic tests should be suggested in patients with a family history of these disorders before administering any pertinent drug that increases the risk of developing all these syndromes.

## Introduction and background

Neuroleptic malignant syndrome (NMS) is a rare illness that results from reactions to neuroleptic medication that is characterized by fever, lead-pipe rigidity, autonomic dysfunction, and altered mental status. It is a life-threatening complication with a 0.2-3.2% incidence in patients receiving neuroleptics [1]. With the new neuroleptic medications, the incidence has decreased to 0.01% to 0.02% [2]. Due to its life-threatening nature, it requires prompt diagnosis and the ruling out of other similar conditions such as serotonin syndrome and malignant hyperthermia. 

Serotonin syndrome (SS) is a life-threatening condition characterized by autonomic instability, mental status alterations, and neuromuscular anomalies. The syndrome is caused by increasing levels of serotoninergic action in the central nervous system. This condition is possibly triggered by therapeutic drugs, intentional self-poisoning, or inadvertent interactions between drugs [3]. On the other hand, malignant hyperthermia (MH) is a potentially fatal inherited muscle membrane disorder that produces a hypermetabolic state due to exposure to inhaled anesthetics [4]. In table 1, we present the main clinical findings of these conditions.

The clinical picture has been well established in the medical literature for these disorders. Nevertheless, the genetics behind these disorders is not discussed much in the literature. As we see in Table 1, the clinical features of these disorders share some similar presentations. For example, altered mental state, increased muscle tone, and autonomic instability (high temperature, diaphoresis, hypertension) can be seen in all these disorders [1,2,3,4]. Because of these similarities, we focus in this review on consolidating the knowledge of the genetics of these disorders and identify and synthesize relevant information about the possible genetic associations of NMS, SS, and MH.

**Table 1:** Clinical features of neuroleptic malignant syndrome, serotonin syndrome and malignant hyperthermia NMS, neuroleptic malignant syndrome; SS, serotonin syndrome; MH, malignant hyperthermia

	NMS	SS	MH
Precipitated By	Dopamine antagonists	Serotoninergic agents	Inhaled anesthetics especially succinylcholine
Onset	Variable, one-three days	Variable, < 12 hours	Minutes to hours
Resolved	nine days	24 hours	Minutes to hours
Mental Status	Stupor, alert, mutism, coma	Agitation, coma, confusion	Agitation
Vital Signs	Hypertension, tachycardia, tachypnea, hyperthermia (> 40°C)	Hypertension, tachycardia, tachypnea, hyperthermia (> 40°C)	Hypertension, tachycardia, tachypnoea, hyperthermia (> 40°C) (can be as high as (46.0°C)
Muscles	'Lead-pipe' rigidity in all muscle groups	Increased tone, especially in lower extremities	Rigor mortis-like rigidity
Reflexes	Hyporeflexia	Hyperreflexia clonus (unless masked by increased muscle tone)	Hyporeflexia
Mucosa	Hypersalivation	Hyper salivation	Normal
Pupils	Normal	Dilated	Normal
Bowel Sounds	Normal or decreased	Hyperactive	Decreased
Skin	Pallor, diaphoresis	Diaphoresis	Mottled Diaphoresis

## Review

Methods

Data was collected from The National Library of Medicine (PubMed) through the use of the following medical subject headings (MeSH) terms with subheading (“Neuroleptic Malignant Syndrome” MeSH AND “Genetics” MeSH, “Serotonin Syndrome” MeSH AND “Genetics” MeSH, “Malignant Hyperthermia” MeSH AND “Genetics” MeSH. Table 2 shows the number of articles collected at first and for the final review of the study.

**Table 2:** Total records and selected records for the review of the article MeSH, Medical Subjects Subheadings

	(“Neuroleptic Malignant Syndrome” [MeSH] AND “Genetics” [MeSH].	“Serotonine Syndrome” [MeSH] AND “Genetics” [MeSH].	“Malignant Hyperthermia” [MeSH] AND “Genetics” [MeSH].
Total Records	42	17	902
Records Selected	3	6	4

Results

Table 3 shows the number of papers collected after applying the inclusion criteria in the following order. 

**Table 3:** Number of papers used after applying the inclusion criteria MeSH: Medical Subject Headings

	(“Neuroleptic Malignant Syndrome” [MeSH] AND “Genetics” [MeSH].	“Serotonine Syndrome” [MeSH] AND “Genetics” [MeSH].	“Malignant Hyperthermia” [MeSH] AND “Genetics” [MeSH]. From 2010-2020
Total records	42	17	195
Inclusion criteria			
Full texts papers	41	17	187
Studies in human	41	12	170
English language	41	12	161
Studies included: (case reports, journal articles, multicenter studies, clinical studies, observational studies, clinical trials).	33	12	144

Inclusion criteria:

1. Full papers

2. Studies conducted in humans

3. Papers published in the English language

4. Study types that include: case reports, journal articles, multicenter studies, clinical studies, observational studies, clinical trials.

A total of 33 papers were collected for NMS, a total of 12 papers for serotonin syndrome, and a total of 144 papers for MH. With this first collection of 189 papers, an initial screening was conducted based on the following exclusion criteria and 43 papers were removed.

Exclusion criteria:

1. Studies involving animals were excluded.

2. Articles without a visible abstract were excluded. 

3. Study types that included: clinical reviews, systematic reviews, meta-analyses.

Each author reviewed the remaining 146 papers and removed 130 papers due to one or more of the following reasons: no possible extraction of data, duplication of the data, or the study outcome was not compatible with the objective of this review. Finally, a total of 17 papers were used for the discussion in this article. 

Discussion

As mentioned above, NMS, SS, and MH share similar clinical features like altered mental state, increased muscle tone, and autonomic instability. It is also notable to mention that drugs also precipitate these conditions [1-4]. For the reasons noted, it could be suggested that these conditions may share a genetic component. We present the main features of each syndrome's genetics and then conclude if there is a genetic association between these conditions.

Genetics of NMS

The occurrence of NMS in some families suggests the possibility of a genetic mechanism underlying this process [5]. Studies have found that the dopamine D2 receptor (DRD2) gene in humans contains TaqIrestriction fragment length polymorphism, which creates the A1 and A2 alleles that leads to an alteration of DRD2 density and function. Studies during postmortem have found that one or two A1 alleles are associated with low DRD2 density in the brain, mostly on the corpus striatum on the caudate region. The risk of developing NMS is 10.5 times higher in A1 allele carriers than in non-carriers. Individuals who are A1 allele carriers show a decrease in dopaminergic activity and glucose metabolism [6,7]. These findings suggest that those who are A1 allele carriers might experience a higher DRD2 blockage by neuroleptic drugs than non-carriers and are more likely to develop NMS. A pharmacogenomic screening test is necessary to adjust the dose of neuroleptic drugs before providing treatment. Lowering the dose of neuroleptic medicines prescribed for patients who are A1 allele carriers may help avoid NMS development [6,7].

Genetics of SS

Recent data have indicated a possible correlation between the occurrence of serotonin‐based adverse drug reactions like SS and the patient's genotype. In particular, it has been suggested that the single nucleotide polymorphism (SNP) T102→C in the receptor subtype 5‐HT2A may be related to a higher rate of adverse drug reactions and treatment discontinuations due to tolerability problems in patients receiving serotonergic drugs [8].

Serotonin2A gene variations may alter receptor numbers, affinity, or signal transductions. Current evidence suggests that homozygous carriers of the C102 allele of the 5‐HT2A gene are at higher risk of developing serotonin‐mediated adverse drug reactions during antidepressant therapy. Survival analysis has shown that among patients treated with paroxetine, there was a positive linear relationship between the number of C alleles and the probability of discontinuation due to adverse events [8]. In a case study reported by Lattanzi et al. [9], serotonin syndrome was induced by phenelzine alone. Genetic analysis revealed that the patient was homozygous for the C102 allele of the 5‐HT2A gene. Contrary evidence is presented by Cooper et al., who failed to find a significant increase in clinically significant cases of SS in individuals having polymorphisms at the T102C locus [10].

Individual variations in serotonin metabolism by CYPs have also been proposed to contribute to SS susceptibility [11,12,13]. One case report describes SS's development in an individual who was taking the selective serotonin reuptake inhibitor (SSRI) paroxetine in the absence of other known serotonergic medications [12]. While paroxetine infrequently causes SS in isolation, this patient was found to have a polymorphism for the CYP2D6 allele, which may have impaired the metabolism of paroxetine and contributed to the development of SS [12]. A similar case report postulated that altered drug pharmacokinetics might have contributed to SS in a patient taking fluoxetine who was found to have a nonfunctioning CYP2D6 genotype, as well as being heterozygous for an allele of CYP2C19 that results in low metabolizing ability [13]. These observations suggest a possible role of 5‐HT2A T102→C SNP in the development of SS. Pharmacogenetic markers may be useful in predicting antidepressant treatment outcome. One should consider any possible implications when initiating antidepressant therapies.

Genetics of MH

MH is a life-threatening pharmacogenomic disorder of the skeletal muscle. It is characterized by muscle contractions and severe hypermetabolic crisis, which occurs as a response to the use of volatile anesthetics or the neuromuscular blocking agent succinylcholine. As mentioned before with serotonin and neuroleptic malignant syndromes, MH also has a pharmacogenetic predisposition leading to increased susceptibility when a patient is in treatment with these medications. In the last three decades, with advances in genetic and molecular technologies, hyperthermia susceptibility (MHS) has been correlated with genetic components such as an inherited autosomal dominant pattern with variable penetrance often difficult to determine [14]. Mutations in genes that code for the ryanodine receptor subtype 1 (RYR1) and alpha one subunit of the dihydropyridine receptor (CACNA1S) have been identified as causative factors to express higher adverse outcomes [15].

In skeletal muscles, Ryanodine receptors (RyR) act as essential cytoplasmic calcium (Ca2+) regulators released from the sarcoplasmic reticulum. Mutations in the RYR1 gene have been related to a possible leaking of RyR in affected individuals and a greater capacity to expel Ca2+ across the t-system membrane continuously [16]. As a result of constant Ca2+ expulsion, dysfunctions in skeletal muscle excitation-contraction coupling complex and life-threatening symptoms have been shown. The significance of the pathogenic nature of the RYR1 mutation in MH is further highlighted by its presence in 70% to 86% of patients with MHS [14]. In contrast, only 1% of patients with MHS have CACNA1S mutation, which plays a vital role in the indirect activation of RYR1 when succinylcholine is administrated [14,17].

Genetic Relation Among NMS, SS, and MH?

We did not find any genetic correlation between these disorders after discussing the genetic associations of each condition. The three conditions can present with life-threatening situations. We suppose there may be a family history as a result of genetic alterations. In that case, it is essential to note the history of familial disposition in these disorders to take any details related to a greater susceptibility of suffering one of these syndromes. If a higher susceptibility is suspected, a pharmacogenetic screening test procedure should be recommended before administering appropriate medications.

## Conclusions

NMS, SS, and MH share some aspects of their clinical presentation, but we did not find any genetic correlation. We discovered that NMS, SS, and MH result from different mutations that increase their receptors' susceptibility when neuroleptics, "serotonin drugs," or anesthetics are administered, respectively. As we found in our research, when patients are carriers for specific mutations, the susceptibility to develop these disorders is increased. Because these syndromes have life-threatening clinical presentations, genetic test studies should be performed before administering any dose of neuroleptic drugs, SSRI, or anesthetics in patients with a history of familial susceptibility to these drugs in order to avoid complications. More research should be done to fill the gaps in knowledge about the possible correlation due to the similar clinical presentation between the three syndromes as an adverse outcome of neuroleptic drugs in some patients.
